# Brief report: attitudes towards Covid-19 vaccination among hospital employees in a tertiary care university hospital in Germany in December 2020

**DOI:** 10.1007/s15010-021-01622-9

**Published:** 2021-05-20

**Authors:** Stilla Bauernfeind, Florian Hitzenbichler, Gunnar Huppertz, Florian Zeman, Michael Koller, Barbara Schmidt, Annelie Plentz, Markus Bauswein, Arno Mohr, Bernd Salzberger

**Affiliations:** 1 Department of Hospital Hygiene and Infectious Diseases, University Hospital Regensburg, Franz-Josef-Strauß-Allee 11, 93053 Regensburg, Germany; 2 Center for Clinical Studies, University Hospital Regensburg, Franz-Josef-Strauß-Allee 11, 93053 Regensburg, Germany; 3 Institute for Clinical Microbiology and Hygiene, University Hospital Regensburg, Franz-Josef-Strauß-Allee 11, 93053 Regensburg, Germany; 4 Center for Pneumology, Donaustauf Hospital, Ludwigstraße 68, 93093 Donaustauf, Germany

**Keywords:** Covid-19, Vaccination, Health care workers, Vaccine hesitancy

## Abstract

Coronavirus disease 2019 (Covid-19) vaccination is essential to fight the pandemic. Health care workers (HCWs) are prioritized to get vaccinated, yet uptake of recommended vaccinations is known to be low in this group. In a tertiary care university hospital with a high number of Covid-19 patients in intensive care, 59.5% of surveyed staff (*N* = 2454) were willing to get vaccinated, 21.4% were unsure and 18.7% refused. Vaccine hesitancy was higher in female, younger and healthy employees without contact to Covid-19 patients; nurses (53.3%) were much less willing to get vaccinated compared to physicians (82.7%).

**Supplementary Information:**

The online version contains supplementary material available at 10.1007/s15010-021-01622-9.

## Introduction

Effective vaccines for SARS-CoV-2 have been rapidly developed. Vaccine acceptance will be a critical issue in mass vaccination. A population wide European survey [1] and an international survey [2] both predicted potential vaccine uptakes of about 70%.

HCWs are at a high risk for infection with severe acute respiratory syndrome coronavirus 2 (SARS-CoV-2) and can be a source of transmission to patients and colleagues. Major hospital outbreaks were reported in 2003 and during the current coronavirus pandemic [3, 4]. Nevertheless, uptake of recommended vaccines among HCWs in general is low [5]. Influenza vaccination coverage among HCWs in Europe is less than 30% [6].

University Hospital Regensburg is a tertiary care hospital and the only university hospital in a region in south-east Germany with a high rate of intensive care beds. Since the beginning of the pandemic, 247 Covid-19 patients have been cared for in the intensive care unit, about one third of them on extracorporeal membrane oxygenation (hospital data, April 2021).

In this context, understanding the local attitudes towards vaccination and identifying potential determinants of vaccine hesitancy are essential to effectively encourage vaccine uptake and protect both staff and patients.

## Methods

We conducted a cross sectional survey to study the attitude of hospital employees towards Covid-19 vaccination from December 12th to 21st, 2020. At this time, the mRNA vaccine BNT162b2 from BioNTech/Pfizer was on the verge of authorization in the European Union as its first Covid-19 vaccine and intended to be used in our institution.

Each hospital employee was offered a paper ticket that provided access to an online survey (Table S1, Supplementary Appendix). A ticket contained both a unique QR-Code and a unique access code for the survey website. The survey could have been completed either with an electronic device via QR-Code-App or by directly accessing the website, thus ensuring anonymity of the participants and preventing multi-use by anti-vaccinationists.

We defined health care workers as all our hospital employees, including clinical administrative staff and further personnel without patient contact or exposure to infectious materials. The hierarchy of vaccine prioritization among different groups of health care personnel is considered in the analysis.

The survey was programmed in REDCap, a web-based clinical data management system. Statistical analysis was performed using Stata 16 (StataCorp). Data are presented as absolute and relative frequencies. Predictors for vaccine acceptance (no vs. unsure and no vs. yes) were analyzed using logistic regression models generating odds ratios (OR). The 95% confidence interval (CI) was used to estimate the precision of the OR.

The study was approved by the local Ethics Committee (Ref. number: 20-2141-101) as well as by the local data protection officer.

## Results

A total of 4861 tickets were distributed. 2454 employees (50.5%) completed the survey. The majority was female (68.0%) and younger than 45 years (57.2%). Most of the participants completed professional training (62.5%), about one-third had a university degree. 23.7% had comorbidities that—according to their self-assessment—put them at an increased risk for a severe course of Covid-19.

25.6% of participants were nurses, 17.2% physicians and 13.7% held other positions with patient contact. 43.4% of participants had no immediate patient contact at work, including administrative, laboratory and technical staff.

49.4% of survey participants reported to be in contact with Covid-19 patients at work. 29.2% indicated an occasional contact, whereas 20.2% had a regular contact defined by working on Covid-19 wards or in the emergency department. (Table 1).

**Table 1:** Participant characteristics

Variable	Total (%) *N* = 2454
Demographic and individual factors	
Age group (years)	
< 25	190 (7.7)
25–34	637 (26.0)
35–44	577 (23.5)
45–54	624 (25.4)
≥ 55	426 (17.4)
Gender	
Male	783 (31.9)
Female	1668 (68.0)
Divers	3 (0.1)
Education	
High school	51 (2.1)
Professional training	1150 (46.9)
Advanced professional training	382 (15.6)
University	869 (35.4)
Risk for a severe course of Covid-19	
No	1501 (61.2)
Unsure	372 (15.2)
Yes	581 (23.7)
Occupational factors	
Occupation	
Nurse	629 (25.6)
Physician	423 (17.2)
Other occupation with direct patient contact*	337 (13.7)
Other occupation without direct patient contact**	1065 (43.4)
Direct contact with Covid-19 patients at work	
No	1242 (50.6)
Occasionally	716 (29.2)
Regularly	496 (20.2)

^*^e.g. physiotherapists, radiological technologists, social workers, dieticians

^**^e.g. laboratory assistance, pharmacists, researchers, administrative staff

During the current influenza season, 54.0% of survey participants received a flu shot, compared to 41.8% during the last influenza season 2019/2020.

As the authorization of BNT162b2, the first Covid-19 vaccine for Europe, was pending, we asked whether employees would be willing to get this vaccine. 59.9% reported yes, 21.4% were unsure and 18.7% refused.

The most important argument for those who refused or were unsure (985 participants) was that the vaccine was not sufficiently tested (780 participants—79.2%). Those bringing forward the argument of insufficient vaccine testing would have accepted a non-mRNA based vaccine in 11.7%, whereas, 47.6% of them hesitated and 40.8% also refused any other vaccine (data not shown).

Among other reasons for refusing were that participants did not feel well informed about the vaccinations (9.9%), regarded themselves as not at risk through Covid-19 (2.7%), reported a history of SARS-CoV-2 infection or had an attitude against vaccinations in general (1.2%, each) (Table 2).

**Table 2:** Attitudes towards vaccination

Variable	Total (%) *N* = 2454
Acceptance of BNT162b2 BioNTech/Pfizer mRNA vaccine	
No	459 (18.7)
Unsure	526 (21.4)
Yes	1469 (59.9)
Reasons for refusal	
Vaccine not sufficiently tested	780 (79.2)
Not well-informed	98 (9.9)
Not at risk through Covid-19	27 (2.7)
History of SARS-CoV-2 infection	12 (1.2)
Attitude against vaccinations in general	12 (1.2)
Fear of injections	6 (0.6)
Other	50 (5.1)
Flu shot in influenza season 2019/2020	
No	1429 (58.2)
Yes	1025 (41.8)
Flu shot in influenza season 2020/2021	
No	1129 (46.0)
Yes	1325 (54.0)

Vaccine acceptance was significantly higher in older age groups, 72.3% of those ≥ 55 years would have taken the vaccine compared to 52.4% of those < 35 years. In univariate logistic regression vaccine acceptance was associated with gender (females were more likely to refuse, OR 0.4, 95% CI 0.3–0.5) and self-assessment of being at increased risk for a severe course of Covid-19 (OR 3.8, 95% CI 2.8–5.3). Considering education, highest acceptance rates were seen in both employees without any professional training (60.8%) and university graduates (74.3%). Consequently, physicians were more likely to accept a Covid-19 vaccine compared to nurses (OR 5.5, 95% CI 3.6–8.5). Another promotive factor was risk exposure. Those who had an occasional (OR 1.4, 95% CI 1.1–1.7) or regular (OR 1.9, 95% CI 1.4–2.5) contact with Covid-19 patients were significantly more likely to accept the vaccine than those without any Covid-19 patient contact. Finally, employees willing to receive flu shots were more likely to accept a Covid-19 vaccine (OR 5.1, 95% CI 3.9–6.6 last season, OR 7.9, 95% CI 6.1–10.1 current season).

Regarding those unsure whether they should get vaccinated against Covid-19 (526 participants), there were no statistically significant differences found in most variables compared to those who completely refused. Only employees who considered themselves at risk for a severe course of Covid-19 and those who had received flu shots during the past two years were significantly more likely to accept the vaccine (Table 3).

**Table 3:** Covid-19 vaccine acceptance

Variable		Covid-19 vaccine acceptance (*N* = 2454) Reference: no acceptance			
	No	Unsure		Yes	
	Total (%) *N* = 459	Total (%) *N* = 526	crude OR (95% CI)	Total (%) *N* = 1469	crude OR (95% CI)
	Demographic and individual factors				
Age group (years)					
< 25	46 (24.2)	63 (33.2)	Reference	81 (42.6)	Reference
25–34	150 (23.5)	135 (21.2)	0.7 (0.4–1.0)	352 (55.3)	1.3 (0.9–2.0)
35–44	113 (19.6)	121 (21.0)	0.8 (0.5–1.2)	343 (59.4)	**1.7** (1.1–2.6)
45–54	112 (17.9)	127 (20.4)	0.8 (0.5–1.3)	385 (61.7)	**2.0** (1.3–3.0)
≥ 55	38 (8.9)	80 (18.8)	1.5 (0.9–2.6)	308 (72.3)	**4.6** (2.8–7.6)
Gender					
Male	90 (11.5)	98 (12.5)	Reference	595 (76.0)	Reference
Female	368 (22.1)	426 (25.5)	1.1 (0.8–1.5)	874 (52.4)	**0.4** (0.3–0.5)
Divers	1 (33.3)	2 (66.7)	1.8 (0.2–20.6)	0	n.d
Education					
High school	10 (19.6)	10 (19.6)	Reference	31 (60.8)	Reference
Professional training	268 (23.3)	313 (27.2)	1.2 (0.5–2.9)	569 (49.5)	0.7 (0.3–1.4)
Advanced professional training	85 (22.3)	76 (19.9)	0.9 (0.4–2.3)	221 (57.9)	0.8 (0.4–1.8)
University	96 (11.1)	127 (14.6)	1.3 (0.5–3.3)	646 (74.3)	**2.2** (1.0–4.6)
Risk for a severe course of Covid-19					
No	360 (24.0)	346 (23.1)	Reference	795 (53.0)	Reference
Unsure	48 (12.9)	82 (22.0)	**1.8** (1.2–2.6)	242 (65.1)	**2.3** (1.6–3.2)
Yes	51 (8.8)	98 (16.9)	**2.0** (1.4–2.9)	432 (74.4)	**3.8** (2.8–5.3)
Occupational factors					
Occupation					
Nurse	142 (22.6)	152 (24.2)	Reference	335 (53.3)	Reference
Physician	27 (6.4)	46 (10.9)	1.6 (0.9–2.7)	350 (82.7)	**5.5** (3.6–8.5)
Other occupation with direct patient contact*	70 (20.8)	85 (25.2)	1.1 (0.8–1.7)	182 (54.0)	1.1 (0.8–1.6)
Other occupation without direct patient contact**	220 (20.7)	243 (22.8)	1.0 (0.8–1.4)	602 (56.5)	1.2 (0.9–1.5)
Direct contact with Covid-19 patients at work					
No	266 (21.4)	284 (22.9)	Reference	692 (55.7)	Reference
Occasionally	125 (17.5)	148 (20.7)	1.1 (0.8–1.5)	443 (61.9)	**1.4** (1.1–1.7)
Regularly	68 (13.7)	94 (19.0)	1.3 (0.9–1.9)	334 (67.3)	**1.9** (1.4–2.5)
Attitude towards vaccination					
Flu shot in influenza season 2019/2020					
No	374 (26.2)	373 (26.1)	Reference	682 (47.7)	Reference
Yes	85 (8.3)	153 (14.9)	**1.8** (1.3–2.4)	787 (76.8)	**5.1** (3.9–6.6)
Flu shot in influenza season 2020/2021					
No	360 (31.9)	304 (26.9)	Reference	465 (41.2)	Reference
Yes	99 (7.5)	222 (16.8)	**2.7** (2.0–3.5)	1004 (75.8)	**7.9** (6.1–10.1)

Significant ORs (95% CI) are presented in bold face; sum row = 100%

## Discussion

Vaccines are regarded as essential to overcome the SARS-CoV-2 pandemic. The development of herd immunity through vaccination depends on virus, vaccine and population factors [7]. Most importantly, people should be willing to get vaccinated. Yet vaccine hesitancy is a growing problem and has been described as a threat to global health in 2019 by the World Health Organization. Its determinants are complex and context-specific and vary across time, place and vaccines [8].

HCWs are at the frontline of Covid-19 response. Vaccination is especially recommended to protect them during occupational exposure and to prevent the spread of the disease.

In December 2020, shortly before the vaccination campaign started, 59.9% of our hospital employees were willing to get vaccinated with BNT162b2, 21.4% were unsure and 18.7% refused.

Covid-19 vaccine acceptability surveys in health care personnel are rare. Those available are from different health care system backgrounds, assess varying groups of HCWs and are conducted at various stages of the pandemic. These are only some factors that make comparison difficult.

During the first wave of the pandemic in March and April 2020 vaccine acceptance rates ranging from 27.7% in Congolese HCWs to 78.1% in Israeli doctors were reported [9].

In a survey among general practitioners and nurses in France and French-speaking parts of Belgium and Canada conducted in October/November 2020, 72.4% were in favor of getting vaccinated [10]. Among HCWs in the United States (staff working in healthcare settings regardless of patient care or contact) surveyed during the same period, 36% were willing to take the vaccine as soon as it became available while 56% were not sure or wanted to wait [11]. Similarly, among Los Angeles HCWs surveyed from September to December 2020, most participants (65.5%) would have delayed vaccination [12].

Receptivity predictors that could be identified in HCWs in our study and elsewhere were perceived risk or exposure and being older, male or a doctor. Previous vaccination history was also associated with acceptance of a Covid-19 vaccine [13]. Regarding influenza vaccination, Hong Kong nurses showed a similar acceptance rate of seasonal influenza vaccination in 2019 and 2020 [14], whereas, our HCWs were much more willing to get vaccinated against influenza this year.

Nurses should be addressed especially in HCW vaccination campaigns. They have a high occupational risk and are often reluctant to get vaccinated. In our hospital, only 53.3% of nurses would have accepted the vaccine compared to 82.7% of physicians. Uncertainty is a big obstacle; information should be provided on safety and efficacy. Furthermore, the value and necessity of immunization should be emphasized.

Hospitals were not successful in achieving high uptakes of recommended vaccinations for HCWs which is especially evident for influenza. By identifying local attitudes and barriers to vaccination, campaigns can be started to strengthen confidence and so increase uptake of Covid-19 vaccines. This survey actually served as first sensitizing our hospital staff for the SARS-CoV-2 vaccination. It was followed by written and audiovisual information from our infectious diseases specialist and virologist presented on intranet. Vaccine acceptance finally exceeded survey results both in physicians and nursing staff (ongoing process, data not shown).

## Limitations

This survey is subject to limitations. It is local data that cannot be applied to other places. It is a snapshot depicting staff’s attitude just before vaccinations started in our hospital. Finally, as we were waiting for BNT162b2, this study is limited to the uptake of an mRNA vaccine with a German company involved in the development. Nevertheless, the present study may serve as a benchmark to set results into perspective from follow-up studies in our center and studies generated in other centers.

## Supplementary Information

Below is the link to the electronic supplementary material.

Supplementary file 1 (DOCX 111 KB)
