# Supplementary material for: Brief report: attitudes towards Covid-19 vaccination among hospital employees in a tertiary care university hospital in Germany in December 2020
Source: Infection. 2021 May 20;49(6):1307–11. doi: 10.1007/s15010-021-01622-9 (PMC8134963; doi:10.1007/s15010-021-01622-9)
Supplement: Supplementary file 1 — (DOCX 111 KB) [file 15010_2021_1622_MOESM1_ESM.docx]

**Supplementary Appendix**

**Table S1. survey**

| **1. What is your gender?** | - male - female - divers |
| --- | --- |
| **2. What is your age group?** | - < 25 - 25-34 - 35-44 - 45-54 - > 55 |
| **3. Where do you work?** | - nursing - physician (with patient contact) - physician (without patient contact) - other post with patient contact - other post without patient contact - administration |
| **4. What is your education?** | - high school - professional training - advanced professional training - university |
| **5. Do you regard yourself as at risk for a severe**  **course of Covid-19?** | - no - yes - unsure |
| **6. Do you have a regular direct contact with Covid-**  **19 patients at work?**  (working in the emergency ward, on Covid-19 intensive  care or Covid-19 regular ward) | - no - yes |
| only if 6 is “no”:  **7. Do you have an occasional direct contact with**  **Covid-19 patients at work?**  (performing examinations or other procedures in Covid-19  patients, e.g. endoscopy, echocardiography, surgery) | - no - yes |
| At University Hospital Regensburg there will be the possibility to get vaccinated against Covid-19 with the mRNA vaccine Comirnaty® from BioNTech/Pfizer. Two doses will be applicated 3 weeks apart.  **8. Would you currently like to get vaccinated?** | - no - yes - unsure |
| only if 8 is “no” or “unsure”:  **8.1.** **What is your main objective against**  **vaccination?** | - the vaccine is not sufficiently tested; effectiveness and side effects are not well evaluated - I had Covid-19 and do not need a vaccination - I am not at risk through Covid-19 - I am afraid of injections - I am against vaccinations in general - I do not feel well informed - other reason |
| only if 8.1 is “other”:  **8.1.1. Here you can write another reason:** |  |
| only if 8.1 is “vaccine is not sufficiently tested”:  **8.1.2. Would you consider a vaccination**  **with a non-mRNA based vaccine?** | - no - yes - unsure |
| 9. Have you received a flu shot in the current influenza  season? | - no - yes |
| 10. Have you received a flu shot in the last influenza  season? | - no - yes |
